# The Application of Stress Modifiers as an Eco-Friendly Approach to Alleviate the Water Scarcity in Ajwain (*Carum copticum* L.) Plants

**DOI:** 10.3390/plants13233354

**Published:** 2024-11-29

**Authors:** Saeid Heydarzadeh, Ahmad Tobeh, Sodabeh Jahanbakhsh, Salim Farzaneh, Ermenegilda Vitale, Carmen Arena

**Affiliations:** 1 Department of Plant Genetics and Production Engineering, Faculty of Agriculture and Natural Resources, University of Mohaghegh Ardabili, Ardabil, P.O. Box 179, Iran; s.heydarzadeh@uma.ac.ir (S.H.);; 2 Department of Biology, University of Naples Federico II, 80126 Naples, Italy; 3 NBFC-National Biodiversity Future Center, 90133 Palermo, Italy

**Keywords:** ajwain plants, nature-based solutions, environmental stress, stress modulators, sustainable agriculture, water deficit

## Abstract

Stress modifiers are recognized as biostimulants providing beneficial effects on various plant species. However, the specific potential of modulators such as melatonin, chitosan, humic acid, and selenium in enhancing the resistance of ajwain (*Carum copticum* L.) plants to water scarcity remains an open question. To address this knowledge gap, we conducted a randomized, field block-designed factorial experiment over two years (2022–2023) to compare the effectiveness of these biostimulants in mitigating the impact of water shortage on ajwain plants. This study involved three irrigation regimes: 100% field water capacity (FC100%—unstressed), 75% irrigation deficit (FC75%—moderate) and 50% irrigation deficit (FC50%—severe), and four modifier treatments (melatonin, chitosan, humic acid, selenium), plus untreated controls. Plant growth, seed yields, essential oil production, as well as eco-physiological traits were studied to assess the efficacy of these compounds as stress modulators. Water regimes and stress modifier applications, as a single factor or in synergy, significantly affected plant physiology and seed yield, highlighting the importance of sustainability in agricultural practices. Compared to FC100%, biological and seed yield, chlorophyll, and nutrient content decreased under FC75% and FC50%, while essential oil production, proline, soluble sugars, flavonoids, phenols and antioxidant enzymatic activity increased. Notably, regardless of the type of modulator used, the application of these modifiers improved all physiological attributes under moderate and severe irrigation deficits. Among the involved compounds, melatonin induced the most pronounced effects, leading to higher biological and seed yield, essential and fixed oil production, relative leaf water content, chlorophyll and nutrient concentration, and antioxidant activity. Our results demonstrate that such compounds effectively function as stress modulators against water scarcity in ajwain plants by preserving specific eco-physiological traits and promoting water saving. These findings provide valuable insights into their use as a nature-based solution for addressing water stress in sustainable agriculture and climate change challenges.

## 1. Introduction

Ajwain (*Carum copticum* L.), a medicinal herb belonging the Apiaceae family, is cultivated in Europe, Iran, India, Egypt, and Pakistan. This plant finds several uses in traditional medicine for its antibiotic and antimicrobial properties deriving from chemical constituents like flavonoids, aromatic oils, phenolics (carvacrol), and terpenes (γ-terpinene) [1]. Pharmacological and clinical trials indicate that *C. copticum* is effective in treating pharyngitis, showcasing benefits like antioxidant, antiparasitic, expectorant, and antilithiatic effects [2]. Considering that many synthetic drugs for the treatment of joint disorders may have adverse effects on organisms, there is a growing interest in the utilization of medicinal herbs containing natural bioactive compounds, such as ajwain, against diverse diseases [3], which, in addition, also shows an elevated resistance to water scarcity. The resistance to water stress is a valuable trait in a plant for counteracting the effect of climate change and saving the wasting water, especially in semi-arid and arid regions usually subjected to limited rainfalls, like some countries such as Iran [4].

Water scarcity threatening the agricultural sector highlights the need to implement sustainable water management strategies [5,6,7,8,9]. Within certain limits, crops can tolerate water deficit by adjusting osmotic potential and activating antioxidant defenses to mitigate damages [10]. Osmotic adjustments are necessary for maintaining turgor pressure and cell structure and function [11]. At the same time, antioxidant mechanisms play a crucial role in scavenging free radicals, thus protecting membranes, proteins, lipids, and genetic material [12,13]. Interestingly, water deprivation can also stimulate secondary metabolism in plants, enhancing the synthesis of many compounds, including antioxidants and essential oils that strengthen plant defenses against stress while improving nutritional value [10,12].

To improve ajwain cultivation, pioneering technologies to maintain water balance under water scarcity conditions have been explored [14]. Among them, applying stress modifiers as biostimulants, offers a natural-based solution [15], playing a pivotal role in crop nutrition [8] and stress resistance, by optimizing plant nutrient and water uptake and moisture retention.

In our study, we focused on specific modulators known for their beneficial effect: melatonin, chitosan, humic acid, and selenium.

Despite the costs associated with purchasing and applying these modulators on crops, their benefits—such as promoting organic farming practices, reducing chemical usage, and enhancing crop yield—can outweigh these expenses [15].

Chitosan (CHN) is a natural biopolymer derived from the detoxification of chitin, which is found in the exoskeletons of arthropods and the cell walls of fungi. It is recognized as the second most sustainable carbon source after lignocellulosic materials [10]. The production of chitosan involves several chemical processes, including demineralization, deproteinization, and deacetylation [10,12]. Chitosan is known to stimulate the synthesis of beneficial secondary metabolites, such as polyphenolics, flavonoids, and lignin while also activating antioxidant enzymes [12]. These effects help mitigate the impacts of biotic and abiotic stresses on plants [14]. Additionally, chitosan exerts several natural properties that enhance the root and exhibits antimicrobial, antifungal, and antiviral activities [10,12,14]. By activating enzymes like phytoalexins and chitinases, it boosts the plant’s resistance to adverse environmental conditions minimizing associated damages [10,12].

Humic acid has been shown to enhance herb growth, particularly under drought conditions, by regulating osmotic pressure and facilitating cell expansion [16]. Additionally, it improves membrane permeability and supports metabolic processes like respiration, photosynthesis, and enzyme activation. Humic acid also serves as a reservoir for essential metals, promoting better microbiota health [17].

Melatonin**,** a molecule rapidly absorbed by plants [11], exhibits a multitude of roles in physiological processes. It promotes herb and plant growth and maturation and enhances photosynthesis and enzymatic antioxidant defenses during water deficit periods [13]. Studies demonstrated that melatonin is a versatile compound, strengthens plant tolerance against a variety of environmental stressors such as extreme temperatures, salinity, chemical compounds, and pathogens [18]. Furthermore, its positive influence on maintaining reactive oxygen species (ROS) homeostasis and antioxidant capacity in drought-stressed herbs such as *Origanum marjorana* L. [11] and *Hibiscus syriacus* L. [19] further confirms its benefits.

Selenium, an ion essential for plant growth and development, acts as a cofactor for several essential enzymes [20]. Its application improves crop yield and quality [21], increases photosynthesis, carbohydrate and secondary metabolite synthesis, and enzymatic antioxidant activity [21]. Additionally, selenium plays a crucial role in delaying plant senescence [22] and promoting plant resistance to drought stress [23,24].

While the beneficial effects of stress modifiers are well established, their impact can vary across different plant species. The potential benefits of melatonin, chitosan, humic acid, and selenium applications in enhancing ajwain growth and its resistance to water scarcity assume considerable interest because of the extensive use of this species as a natural medicine in many countries. We conducted a comprehensive experiment over two consecutive years to assess plant performance, focusing on plant growth attributes, essential oil production, and physiological response as main parameters. Our findings provide valuable insights into using these compounds as environmentally friendly practices for sustainable agriculture.

## 2. Results

### 2.1. Two-Year Effect of Water Treatments and Stress Modifier Applications on Biological and Seed Yield and Nutritional Traits of Ajwain Plants

#### 2.1.1. Biological and Seed Yield

We analyzed the impact of different stress modifiers on ajwain plants under limited water conditions using an irrigation regime (Ir) and a modifier application (M) as independent variables, as well as their interaction (Ir × M) over a two-year period. Our findings indicate that both the irrigation regime (Ir) and type of modifier (M), along with their interaction, had a significant impact on all parameters. Additionally, the sampling year (Y) independently influenced biological and seed yield, fixed and essential oil yield, as well as potassium, nitrogen, and phosphorus leaf content, without any interaction with other variables.

**Figure 1:**
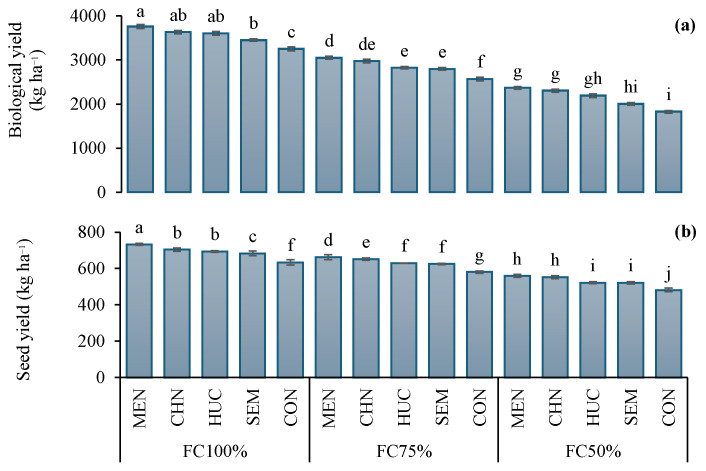
Interaction effect of stress modifier applications (MEN, melatonin; CHN, chitosan; HUC, Humic acid; SEM, Selenium; and CON, control) and irrigation regimes (FC100%, FC75%, and FC50%) on biological yield (**a**) and seed yield (**b**) of ajwain plants. Different letters indicate statistically significant differences by LSD test at *p* < 0.05.

The average biological yield (BY) and seed yield (SY) in 2022 were 2758 and 601.6 kg ha^−1^, while, in 2023, they were 2690 and 630 kg ha^−1^, respectively (Table 1). The highest biological yield and seed yield 3405 and 693.5 kg ha^−1^, respectively, were observed in plants grown under FC100% (Table 1). All treatments enhanced the biomass yield and seed yield compared to control. Melatonin determined the highest increase in both parameters (Table 1). The interaction Ir × M significantly affected biological yield and seed yield (Table 1). In particular, all modifiers increased the biomass yield and seed yield at all irrigation treatments compared to the control (Figure 1a,b). The highest values of biomass yield (3762 kg ha^−1^) and seed yield (733 kg ha^−1^) were found in plants treated with melatonin under FC100% (Figure 1a,b), while the lowest ones (1476 and 482 kg ha^−1^, respectively) were detected in control plants under FC50% (Figure 1a,b).

#### 2.1.2. Fixed Oil and Fixed Oil Yield, Essential Oil and Essential Oil Yields

**Figure 2:**
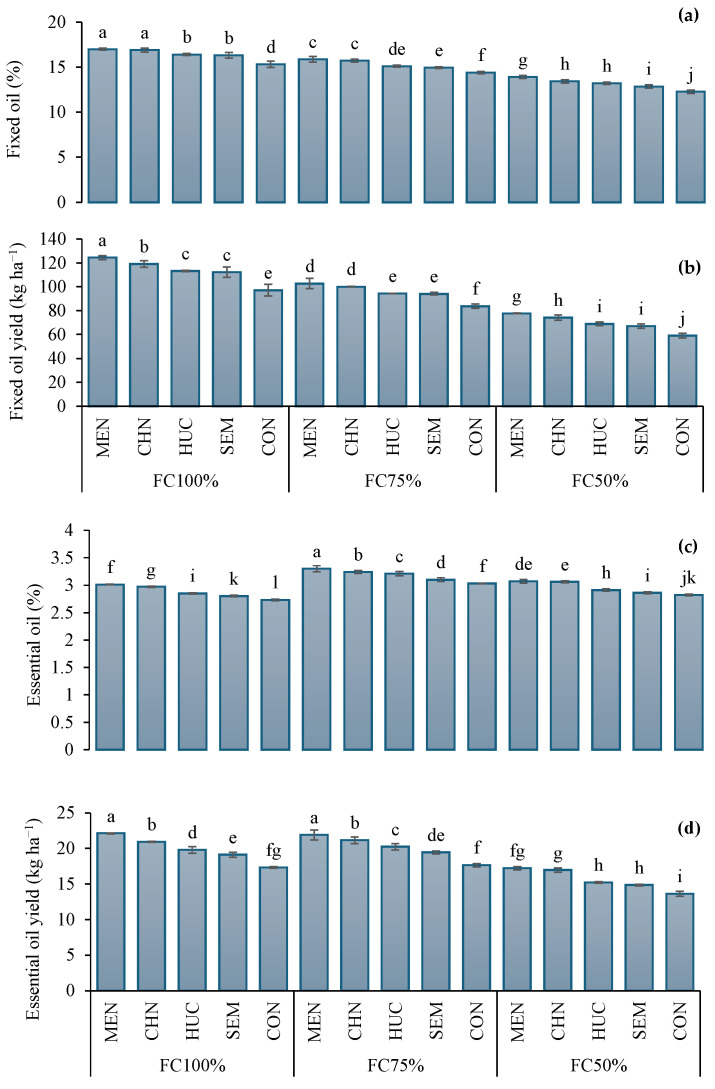
Interaction effect of stress modifier applications (MEN, melatonin; CHN, chitosan; HUC, humic acid; SEM, selenium; and CON, control) and irrigation regimes (FC100%, FC75%, and FC50%) on fixed oil (**a**) and fixed oil yield (**b**), essential oil (**c**) and essential oil yield (**d**) of ajwain plants. Different letters indicate statistically significant differences by LSD test at *p* < 0.05.

The average fixed oil (FO) and fixed oil yield (FOY) were 14.9% and 90.8 kg ha^−1^ in 2022 and 14.9% and 94.9 kg ha^−1^ in 2023 (Table 1). Under FC100%, the fixed oil percentage and fixed oil yield concentration were higher compared to FC75% and FC50%, and the greatest values were obtained with the application of melatonin (Table 1). More specifically, fixed oil reached 16.9%, while fixed oil yield was 124 kg ha^−1^. Conversely, the lowest values (fixed oil 12.3% and fixed oil yield 59.1 kg ha^−1^) were recorded in control plants at FC50% (Figure 2a,b).

The average essential oil yield (EOY) was higher in 2023 (18.9 kg ha^−1^) than 2022 (18.1 kg ha^−1^) (Table 1). Notably, plants subjected to FC75% and FC50% treatments showed higher percentages of essential oil (EO) compared to respective samples at FC100% (Table 1). Among biostimulants, melatonin favored the highest essential oil and essential oil yield (Table 1). Specifically, the highest essential oil (3.30%) was observed in plants treated with melatonin and subjected to moderate water stress (FC75%), while the highest essential oil yield (22.1 kg ha^−1^) was recorded in melatonin plants receiving moderate stress (FC75%) or normal irrigation (Figure 2c,d). Conversely, the lowest values of essential oil (2.73%) and essential oil yield (13.6 kg ha^−1^) were measured in control plants under FC100% (Figure 2c), and FC50% (Figure 2d), respectively.

#### 2.1.3. Nitrogen, Phosphorus, and Potassium Leaf Concentration

**Figure 3:**
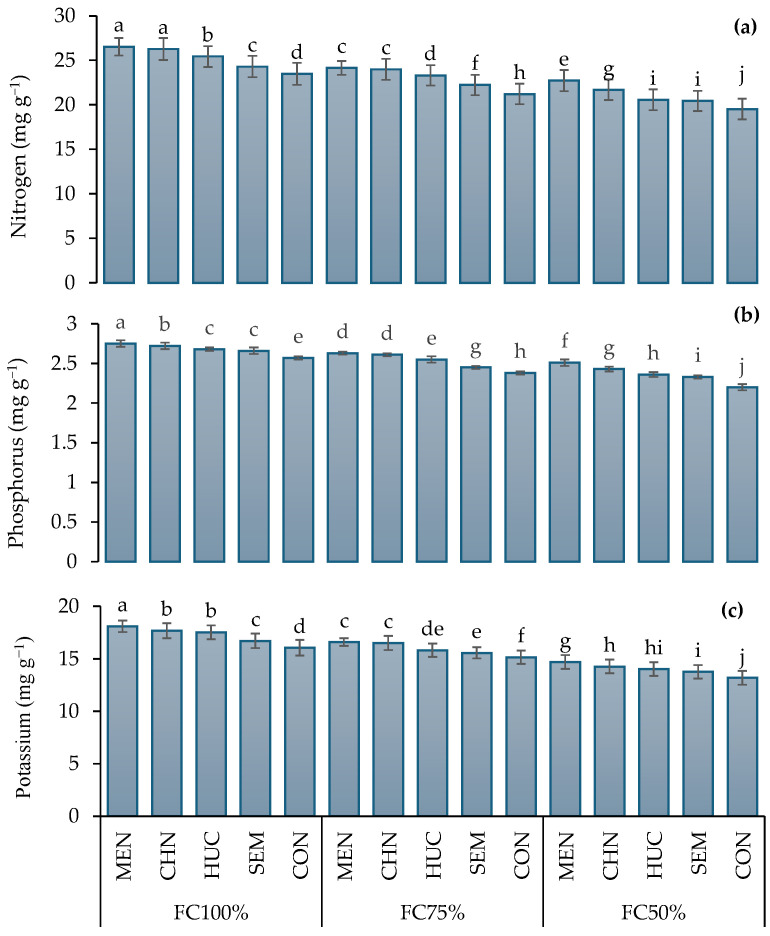
Interaction effect of stress modifier applications (MEN, melatonin; CHN, chitosan; HUC, humic acid; SEM, selenium; and CON, control) and irrigation regimes (FC100%, FC75%, and FC50%) on nitrogen (**a**), phosphorus (**b**), and potassium (**c**) of ajwain plants. Different letters indicate statistically significant differences by LSD test at *p* < 0.05.

Leaf nitrogen, phosphorus, and potassium concentration were significantly higher in 2023 compared to 2022 (Table 1). Leaves of plants grown under FC100% displayed levels of nitrogen, phosphorus, and potassium that were significantly higher than plants grown under FC75% and FC50% (Table 1). Among biostimulants, melatonin application induced the highest nitrogen, phosphorus, and potassium concentrations (Table 1), with maximum values (26.5, 2.75, and 18.1 mg g^−1^, respectively) under FC100% (Figure 3a–c). At FC100%, in plants treated with melatonin and chitosan, no significant difference was observed in leaf nitrogen content while at FC75% in leaf phosphorus concentration (Figure 3a,b). The lowest nitrogen, phosphorus, and potassium levels (19.5, 2.20, and 13.2 mg g^−1^, respectively) were recorded in control plants under FC50% (Figure 3a–c).

### 2.2. Two-Year Effect of Water Treatments and Stress Modifier Applications on Eco-Physiological Traits and Antioxidant Defenses of Ajwain Plants

A two-year experiment conducted in the field showed that the eco-physiological traits and antioxidant defenses of ajwain plants were significantly affected by the water treatment (Ir) and stress modifier application (M) as main factors and by the interaction (Ir × M) (Table 2). Among considered variables, year (Y), as a main factor, influenced only relative water content, chlorophyll, and total soluble sugar content, as well as total flavonoid and polyphenol concentrations.

#### 2.2.1. Leaf Relative Water Content, Chlorophyll a and b Concentrations

**Figure 4:**
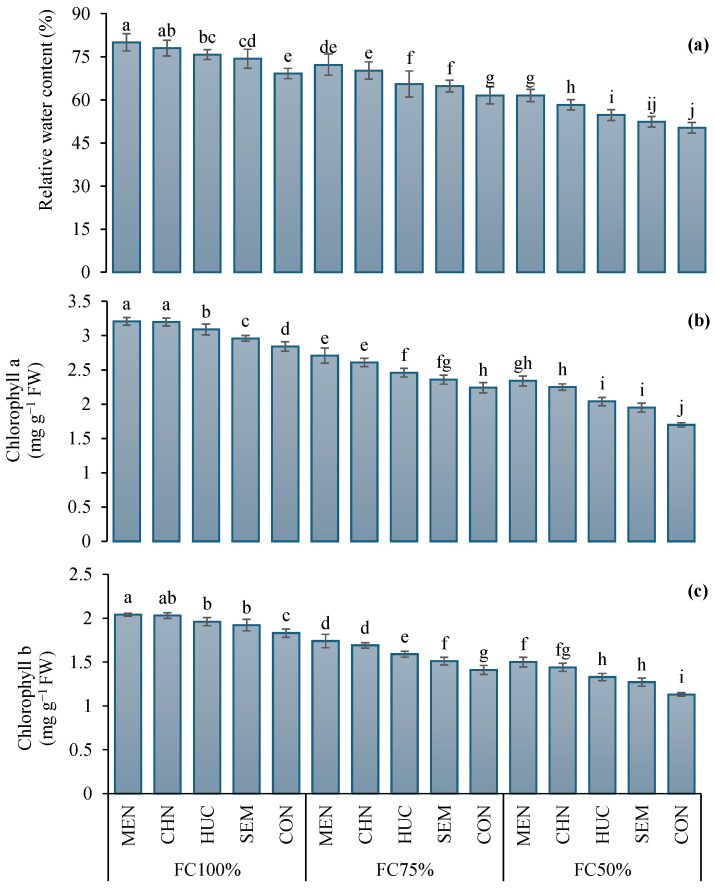
Influence of stress modifier applications (MEN, melatonin; CHN, chitosan; HUC, humic acid; SEM, selenium; and CON, control) and irrigation regimes (FC100%, FC75%, and FC50%) on relative water content, RWC (**a**), chlorophyll a (**b**) and chlorophyll b (**c**) concentrations of ajwain plants. Different letters indicate statistically significant differences by LSD test at *p* < 0.05.

The leaf relative water content (RWC) in 2023 was significantly higher than in 2022 (67.6% and 64.3%, respectively) (Table 2). Plants at FC 100% showed higher relative water content values compared to plants grown under FC75% and FC50% (Table 2). The interaction Ir × M significantly influenced the relative water content (Table 2). In particular, the highest value (80%) was recorded in plants treated with melatonin under FC100%, while the lowest (50.3%) was observed in control plants at FC50% (Figure 4a).

Leaf chlorophyll a (Chl a) and b (Chl b) concentrations were higher in 2023 than 2022 with values on average of 2.46 and 1.59 mg g^−1^ of FW in 2022, and 2.57 and 1.66 mg g^−1^ of FW in 2023, respectively (Table 2). Under FC100%, plants exhibited higher levels of chlorophyll a and b than FC 75% and FC 50% (Table 2). The use of stress modifiers, regardless of the type of molecule, significantly increased both chlorophyll a and b concentration compared to the control (Table 2). The application of melatonin under the FC 100% regime induced the highest amount of chlorophyll a and b (3.21 and 2.04 mg g^−1^ of FW, respectively) (Figure 4b,c). Conversely the lowest values of chlorophyll a and b (1.70, and 1.13 mg g^−1^ of FW, respectively) were detected in control plants under FC50% (Figure 4b,c).

#### 2.2.2. Osmotic Concentration

**Figure 5:**
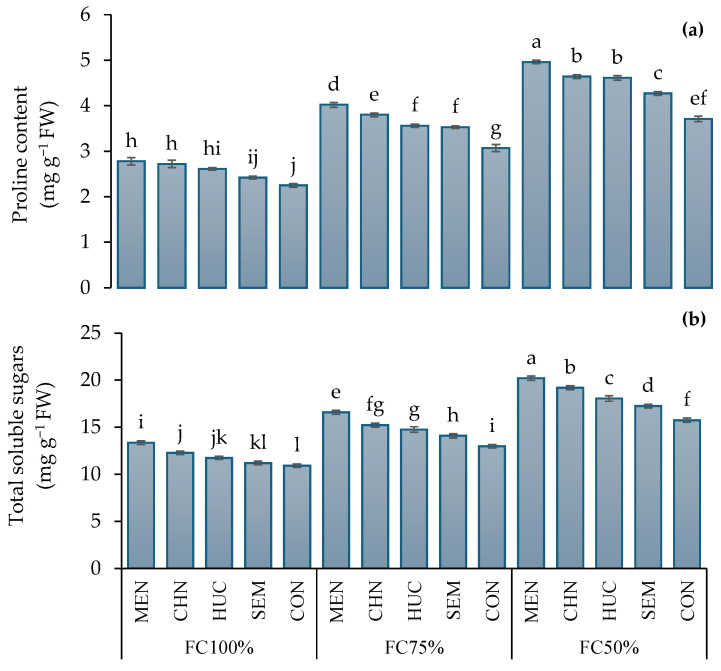
Effect of stress modifier applications and (MEN, melatonin; CHN, chitosan; HUC, humic acid; SEM, selenium; and CON, control) and irrigation regimes (FC100%, FC75%, and FC50%) on proline (**a**) and total soluble sugars (**b**) of ajwain plants. Different letters indicate statistically significant differences by LSD test at *p* < 0.05.

Proline concentration (Pro) did not change between 2022 and 2023, showing values on average of 3.56 and 3.50 mg g^−1^ of FW, differently from total soluble sugar concentration, which was lower in the plants harvested in 2022 compared to 2023 (15.6 vs. 14.2 mg g^−1^ of FW, respectively). Under FC 75% and FC 50%, proline and total soluble sugars were significantly higher compared to FC 100% (Table 2). Among stress modifiers, melatonin induced an increment of proline and total soluble sugars (TSSs) (Table 2), particularly under FC50% with peaks of 4.96 and 20.2 mg g^−1^ of FW, respectively (Figure 5a,b), while the lowest concentrations (2.25 and 10.9 mg g^−1^ of FW, respectively) were detected in control plants under FC100% (Figure 5a,b).

#### 2.2.3. Antioxidant Enzyme Activity

**Figure 6:**
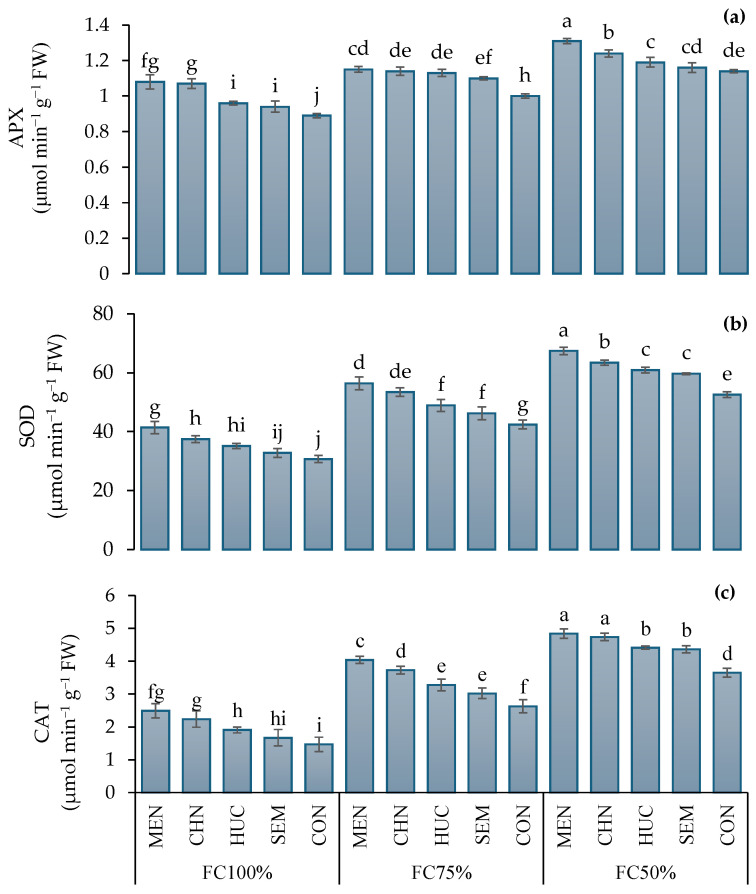
Influence of stress modifier applications (MEN, melatonin; CHN, chitosan; HUC, humic acid; SEM, selenium; and CON, control) and irrigation regimes (FC100%, FC75%, and FC50%) on APX, ascorbate peroxidase activity (**a**); SOD, superoxide dismutase activity (**b**); and CAT, catalase activity (**c**) of ajwain plants. Different letters indicate statistically significant differences by LSD test at *p* < 0.05.

The ascorbate peroxidase (APX), superoxide dismutase (SOD), and catalase (CAT) antioxidant activity were similar in the plant extracts harvested in 2022 and 2023 (Table 2). In response to different irrigation regimes, ascorbate peroxidase, superoxide dismutase and catalase activities were significantly higher under FC75% and FC50% compared to FC100% (Table 2). Among biostimulants, the treatment of ajwain plants with melatonin induced the highest ascorbate peroxidase, superoxide dismutase, and catalase scavenger activities (Table 2) that peaked under FC50%, reaching values of 1.31, 67.4, and 4.84 μmol min^−1^ g^−1^ of FW, respectively (Figure 6a–c). The lowest values of APX, SOD, and CAT (0.89, 30.7, and 1.47 μmol min^−1^ g^−1^ of FW, respectively) were recorded in selenium and control plants under FC100% (Figure 6a–c).

#### 2.2.4. Total Phenol and Flavonoid Content

**Figure 7:**
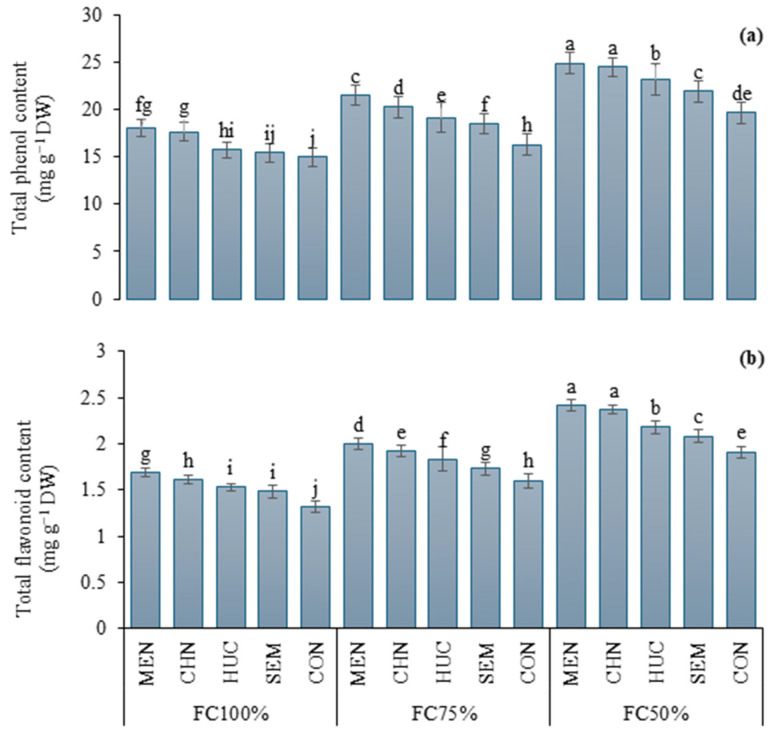
Effect of stress modifier applications (MEN, melatonin; CHN, chitosan; HUC, humic acid; SEM, selenium; and CON, control) and irrigation regimes (FC100%, FC75%, and FC50%) on total phenol content (**a**) and total flavonoid content (**b**) in ajwain plants. Different letters indicate statistically significant differences by LSD test at *p* < 0.05.

Total flavonoid content (TFC) and total polyphenol content (TPC) of ajwain plants was significantly lower in 2023 compared to 2022 (Table 2). Among biostimulants, melatonin and chitosan induced the highest total flavonoid and polyphenol content (Table 2). However, all modifiers significantly increased the flavonoid and polyphenol concentration compared to control regardless of irrigation regimes (Figure 7a,b). The highest total flavonoid and polyphenol content (24.9 and 2.41 mg g^−1^ of DW, respectively) were found in plants treated with melatonin and chitosan under FC50% (Figure 7a,b); in contrast, the lowest flavonoid and polyphenol level (15.0 and 1.32 mg g^−1^ of DW, respectively) was recorded in control plants under FC100% (Figure 7a,b).

## 3. Discussion

Water scarcity significantly inhibits plant growth by affecting structural, biological, and molecular characteristics and by impacting physiological processes such as nutrient absorption and photosynthesis [25]. To enhance the tolerance of ajwain (*Carum copticum* L.) to water scarcity, we treated the plants with stress modifiers [16,20,26], namely melatonin, chitosan, humic acid, and selenium. We applied different water regimes to both treated and untreated plants to evaluate the effectiveness of these treatments under mild and severe water scarcity conditions. Specifically, we selected 50% and 75% field capacity (FC) levels to develop sustainable water management strategies for Iranian farmers, who typically operate within this range to face the ongoing water challenges [27,28,29,30]. The 100% FC level was used as a control to provide adequate moisture, allowing for comparison with traditional over-irrigation practices.

### 3.1. Effect of Stress Modifier Application on Essential Oil Attributes, Leaf Nutritional Status, and Water Relationships

Our study reveals a significant decrease in both biological and seed yield in untreated plants experiencing mild to severe water scarcity. This decline is likely due to a reduction in photosynthetic carbon gain, which subsequently shortens the growth period and limits the effective seed-filling time. As a result, there is a reduced production and transfer of photosynthates to the seeds [1]. Indeed, the water shortage hampers moisture and nutrient absorption inducing roots to require more energy for resources’ uptake; it also compromises hormone production and enzyme activity, as well as photosynthetic efficiency, growth, and yield [1,10], although increased essential oil may partially offset it [31,32,33]. However, the foliar application of melatonin, chitosan, humic acid, and selenium improved both biological and seed yield compared to untreated samples across all water regimes by promoting osmotic regulation and enhancing the activity of antioxidant enzymes, chlorophyll content, Rubisco concentration and ATP and NADPH production [15]. These modifications determined positive feedback on carbon assimilation rates of whole plant [10,13,24]. Notably, melatonin showed the most significant effect among modifiers, according to results reported in Rafique et al. [34], who demonstrated the effectiveness of this compound on *Brassica napus* growth, seed productivity, and resistance to water stress.

Plants typically counteract water deprivation by adjusting leaf gas exchanges, closing stomata, reducing transpiration, and increasing water use efficiency. However, such strategies can limit CO_2_ intake and impair Rubisco carboxylation activity, negatively affecting oil grain yield [33,35,36]. The foliar application of melatonin, chitosan, humic acid, and selenium increased the biological yield of ajwain plants, even in conditions of severe water scarcity. This improvement may be due to higher leaf relative water content, driven by greater proline and soluble sugar levels resulting from combining these modifiers and water stress. Indeed, previous studies have shown that such modifiers promote leaf area expansion and optimize light harvesting and utilization in photosynthesis [15]. They also influence plant metabolism by activating phytohormone synthesis, enhancing leaf water retention, gas exchanges, mineral transport, and nutrient uptake [17,20,37,38,39]. Accordingly, our findings indicate that treated plants have higher concentrations of nitrogen, phosphorus, and potassium in leaves than control plants, confirming the role of these biostimulants in favoring nutrient translocation and utilization.

A valuable attribute of ajwain plants is their richness in essential oils. The yield of fixed oil in plants depends on the fixed oil percentage and seed yield, and, consequently, any factor influencing these two attributes also affect the overall oil yield. Our study demonstrated that, under moderate and severe irrigation deficits, the essential oil content and yields decrease compared to 100% water field capacity, but the application of the stress modifiers, and, in particular, melatonin, emphasize the essential oil production and yield compared to untreated plants subjected to limited water supply.

Our data also provided evidence that, under water shortage, ajwain plants produced more antioxidant molecules and increased phytochemical production. These results are consistent with other studies, which demonstrated the role of biostimulants in enhancing the production of bioactive compounds during stress, ultimately improving seed oil yield and quality [40,41,42,43,44,45,46]. Additionally, the elevated levels of abscisic acid produced during water scarcity help plants to manage grain desiccation and promote lipid storage and fatty acid synthesis in grain embryos [47]. Carbohydrates transported to seeds further increase the oil content, as they are precursors for fatty acid production [48]. Our research demonstrated that ajwain plants achieved the highest essential oil yield under moderate stress, indicating the seeds’ potential for industrial use. Notably, water scarcity increases the density of essential oil-secreting glands, enhancing overall essential oil production [41,49], which also act as protectants against environmental stress [50].

The synthesis of essential oils, classified as terpenoids, require acetyl-CoA, and end products of the light phase of photosynthesis, namely, NADPH and ATP [51], as well as nutrient availability [52,53]. Notably, even during water scarcity, stress modifiers, especially melatonin, helped maintain essential macronutrient concentration (nitrogen, phosphorus, and potassium) in leaves compared to untreated plants, enhancing plant resilience under unfavorable conditions [2,51].

Foliar applications of stress modifiers stimulate growth hormones like cytokinins and boost pathways that produce secondary metabolites, including those responsible for essential oil synthesis in flowers and leaves, as well as antioxidant molecules [2,52,53]. Our data demonstrated that stress modifiers strengthen plant defenses and enhance plant–soil water relationships, even under water scarcity.

Plants treated with stress modifiers, especially melatonin, showed a higher leaf water content than untreated ones. This property is an indicator of improved water balance likely ascribed to a better water uptake by roots, the expansion of which is favored by the biostimulant application [8,50,51].

### 3.2. Effect of Stress Modifier Application on Leaf Photosynthetic Pigments, Osmotic Concentration, and Antioxidant System

In plants subjected to moderate and severe irrigation deficits, the concentration of chlorophyll a and b significantly declined compared to unstressed plants irrespectively from stress modifier treatments. It is reasonable to hypothesize that water deficits may have compromised the enzymatic pathway involved in pigment synthesis, increasing chlorophyllase activity and reactive oxygen species production [33,34,41,54]. However, despite the overall pigment reduction under water scarcity, all plants treated with different stress modifiers exhibited higher chlorophyll concentration compared to untreated plants, suggesting a putative role of modifiers in contrasting the ROS action and maintaining membrane and protein integrity under stress. Studies conducted on melatonin and chitosan demonstrated that their application enhanced the expression of ROS-scavenging enzymes, reduced pigment breakdown, and led to notable increases in chlorophyll concentrations, also delaying the leaf senescence [18,55].

During drought stress or in conditions of limited water supply, plants also prioritize the production of osmoregulatory molecules, such as soluble sugars (i.e., sucrose, fructose), glycine betaine, proline, secondary metabolites, and essential oils, which protect against environmental stresses [25,33,47,49].

Plants treated with modifiers, such as melatonin and chitosan, experienced significant increments in total soluble sugars compared to untreated ones, likely due to a higher α-amylase activity, which breaks down starch into sugars and reduces exportation to roots [50,56,57]. It has been demonstrated that these stress modifier molecules are also involved in H_2_O_2_ removal, quenching the accumulation of ROS, and preventing stomatal closure [53,58].

Proline, alongside trehalose, serves as an osmoprotectant during drought, enhancing cellular integrity and safeguarding cytosolic enzymes [1,7,42,51]. Melatonin and chitosan application promoted osmotic adjustments within ajwain plants, increasing total soluble sugars, proline, and flavonoids, ultimately boosting the resilience under moderate and severe irrigation deficits compared to untreated controls. The high proline levels found following treatments with stress modifiers may derive from reduced glutamate oxidation, increased protein turnover, or modified protein utilization [18,22,37,50,56].

Biostimulant-treated plants under moderate and even more severe water scarcity also exhibited increased activities of antioxidant enzymes, such as ascorbate peroxidase, superoxide dismutase and catalase, compared to controls. It has been demonstrated that selenium, acting as a cofactor, may enhance the activity of antioxidant enzymes, fortifying plant defenses against drought stresses in wheat [21,23]. A similar effect is observed in other crops following melatonin and chitosan application. These biostimulants play a crucial role in reducing lipid peroxidation during water scarcity, thereby minimizing oxidative stress by stabilizing intercellular ROS levels and protecting membranes from drought damage [18,25,54,55,56,57,58,59,60]. The structural properties of melatonin and chitosan, with their accessible amine and hydroxyl groups, enable them to neutralize free radicals [18,25]. Their potential role in influencing specific stress-related gene expression further highlights their importance in improving plant resilience in adverse conditions [60].

Foliar melatonin, chitosan, and humic acid selenium applications also increased total phenolic content and flavonoids across all irrigation regimes, triggering a strong non-enzymatic antioxidant response [49,51]. According to previous findings, polyphenols act as antioxidants, preserving cellular structures and membranes from lipid peroxidation [33,38,49]. Moreover, these biostimulants increase hydrocarbon levels and enzyme activity related to starch biosynthesis and the production of secondary metabolites [28,33,38]. The rise in hydrocarbon facilitates the synthesis of phenolic compounds, particularly flavonoids, by directing more carbon resources to the shikimate pathway, thus enhancing plant tolerance to biotic and abiotic stresses [32,35]. In stressful conditions, higher levels of total phenols and flavonoids result from activating phenyl-alanine ammonia-lyase (PAL) in the phenylpropanoid pathway, essential for maintaining cellular redox balance [25,50]. Our results emphasize the value of stress modifiers in the sustainable cultivation of ajwain plants because their application not only improves plant resistance against water stress but also enhances essential oil yields and the production of secondary metabolites, such as polyphenols.

The demand for polyphenols and essential oils on the food market is high and grows every year; thus, the application of stress modifiers used in our study, and, in particular, melatonin, improved ajwain nutritional and therapeutic properties, resulting in a more valuable final product.

## 4. Materials and Methods

### 4.1. Experimental Setup

A field experiment was performed in the two consecutive years 2022–2023 in the Babelan field (38°16′39″ N, 48°14′32″ E, and altitude of 1354 m) at the University of Mohaghegh Ardabili in Ardabil, northwest Iran (37°45′ to 39°42′′ north latitude and 47°30′ to 48°55′ east of the northwest of Greenwich). The experimental design was factorial, using a complete randomized block design with three replicas per investigation. The factors we considered were the stress modulator treatment obtained by the foliar application of melatonin (MEN), chitosan (CHN), humic acid (HUC), selenium (SEM), and water, as control (CON), and the selection of three irrigation regimes: field water capacity (FC) 100% (FC100%) as control, and FC75% and FC50% as moderate and severe deficit irrigation, respectively. The specific irrigation regimes FC75% and FC50% were properly selected because they align with water management strategies adopted for sustainable agricultural practices in Iran. Therefore, based on the current farming scenarios, FC50% was used to simulate a severe drought, while FC 75% a moderate water scarcity, commonly applied by farmers to manage water saving without excessive losses on crop yield [27,28,29].

The three levels of irrigation regime were applied at the beginning of flowering by controlling water application. Soil moisture content in each plot was monitored daily using a Time Domain Reflectometry device (TRIM-FM TDR 10776, Weilheim, Germany).

At both regions, TDR tubes were placed in each plot to determine soil moisture content in top layer of soil (0–30 cm). The maximum percentage of the allowable depletion of available soil water (ASW) in the effective root zone was determined using Equation (1) [61]:

MAD = FC − Ө/FC − PWP(1)

where MAD is the maximum allowable depletion, FC is the soil volumetric moisture at field capacity, θ is the soil volumetric moisture, and PWP is the soil volumetric moisture at permanent wilting point. The required volume of water (Vd, mm) was estimated according to Equations (2) and (3) [61]:

ASW = FC − PWP(2)

Vd = MAD × ASW × Rz × 10(3)

where the ASW is equal to 15.67 cm m^−1^, Rz the effective rooting depth (0.4 m), and 10 is the conversion constant from cm to mm. The furrow system was used for irrigation. Each plot was watered individually, and a hose (4 cm in diameter) with a gauge was used to adequately convey the required volume of irrigation water.

**Figure 8:**
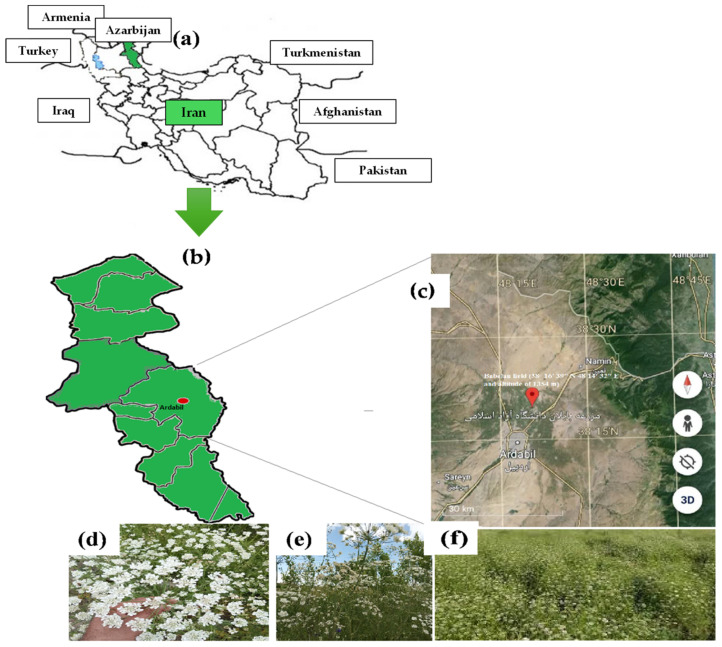
Geographical context of the study area, Ardabil province in the northwest of Iran (**a**,**b**); Babelan field at the University of Mohaghegh Ardabili, Iran (**c**). The image was retrieved from Google Earth (Map data ©2023 Google: Image ©2023); Details of ajwain cultivations in the field (**d**–**f**).

**Figure 9:**
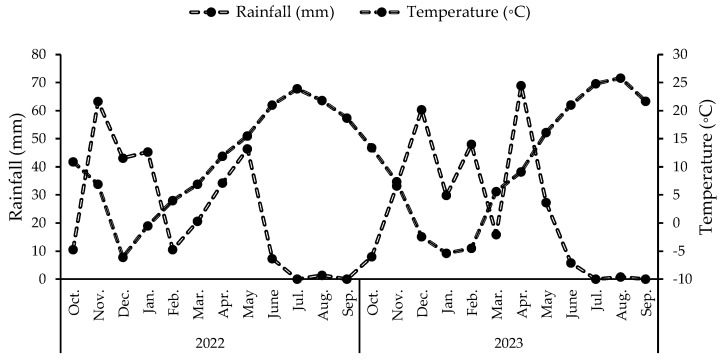
Total rainfall (mm) and average monthly air temperature (°C) for the 2022 and 2023 growing seasons.

The geographical context of the study area was depicted in Figure 8, which depicts the ajwain cultivations. The rainfall and temperature regime during the experiment is reported in Figure 9.

### 4.2. Soil Classification, Plant Material, and Stress Modulators Application

The soil was classified as a silty clay (36% Silt, 33% Clay, 31% Sand) with an electrical conductivity of 0.37 dS m^−1^, determined in 1:2:5 soil–water suspension [62,63], pH 7.8, measured by a pH meter according to Walkley and Black [64], total N 0.11%, analyzed by the Kjeldahl method [64]. Measurements of 10.04 and 344 mg kg^−1^ of available phosphorus (P) and potassium (K) concentration, respectively, were determined according to Olsen [65], and the flame photometer was measured as described in Rowell [66]. After soil classification, the experimental trial was preventively prepared by plowing, disking, and flattening the soil. All plots consisted of 5 rows of herbs spaced 20 cm from each other, with plants spaced 40 cm apart within each row. Seeds of ajwain were sown 20 cm apart within each row in March, in 2022 and 2023. The flowers of ajwain are self-fertile but cross-pollination occurs through insects. During the ajwain growth stage, the foliar spraying of chitosan (0.2 g L^−1^; Sigma-Aldrich Co., Steinheim, Germany), selenium (0.2 g L^−1^; Sigma Aldrich Co., Madrid, Spain), humic acid (0.2 g L^−1^; Acadian AgriTech Co., Dartmouth, Canada), and melatonin (0.2 mg L^−1^; Sigma Aldrich Co., St. Louis, MO, USA) was applied to the leaves in three stages at ten-day intervals, coinciding with periods of moisture stress. Control plants were sprayed with pure water. In detail, melatonin was first dissolved in the minimum amount of ethanol, and then in water, with no adjuvant addition [67,68]. Humic acid was dissolved in distilled water, with no adjuvant addition [48]. Chitosan was dissolved in 1% acetic acid and then diluted with distilled water [69]. The pH of chitosan solutions was adjusted to 6.5 with 1% NaOH [64]. Extra selenium was supplied as granules of sodium selenite (Na_2_SeO_3_). As selenium is insoluble in water, for preparation, it was dissolved before in ethyl alcohol and then in distilled water [70].

Soil characterization was carried out collecting samples at 0–30 cm depth. Leaf samples were collected from different plots at the full flowering stage, corresponding to the developmental stage at which medicinal plants provide the most essential compounds when water stress is applied. Samples were flash-frozen in nitrogen solution and placed at −80 °C for the analyses.

### 4.3. Oil Content and Yield Determination

Samples of each trial treatment were collected separately at the maximum maturing stage, for seed and biological yield. An area of 2 m^2^ within each plot was considered. Ten plants were randomly handpicked from each plot to determine yield traits. Samples were then oven dried at 72 °C for 2 days and weighed to determine the dry weight.

Determination of Fixed Oil Content and Fixed Oil Yield, Essential Oil and Essential Oil Yield in Seeds

The dried seeds were ground into a fine powder to extract oil, following the method reported by the American Oil Chemists’ Society (AOCS, 1993) [71]. Briefly, 5 g seeds were subjected to a 6 h extraction using 300 mL of n-hexane in a Soxhlet ex-tractor at the temperature of 69 °C. Then, the solvent was removed from the extracted oil using a rotary evaporator (Heidolph, Schwabach, Germany). The extracted oil was collected in a dedicated glass container to facilitate further compound isolation and identification [72]. Seed oil content and oil yield were determined as follows [73]:

(4) $$\mathrm{Fixed}\;\mathrm{oil}\;\mathrm{content}\;\left(\%\right)=\frac{\mathrm{Extracted}\;\mathrm{oil}\;\mathrm{content}\;\left(g\right)\;}{5\;g\;\mathrm{of}\;\mathrm{ajwain}\;\mathrm{seed}}\times 100$$

(5) $$\mathrm{Fixed}\;\mathrm{oil}\;\mathrm{yield}\;({\mathrm{kg}\;\mathrm{ha}\;}^{-1})=\;\mathrm{Fixed}\;\mathrm{oil}\;\mathrm{content}\;\left(\%\right)\times \mathrm{seed}\;\mathrm{yield}\;({\mathrm{kg}\;\mathrm{ha}\;}^{-1})$$

The essential oil content of ajwain plants was determined following the Clevenger method [74], using water distillation and an essential oil extraction device. For each sample, 50 g of air-dried ground ajwain seeds were weighed and sieved through a 1 mm screen. The powdered seeds were placed in a jar containing 500 mL of water and boiled in the Clevenger for 3 h to extract the essential oil (EO). Next, the extracted essential oil was weighed (g), and its amount and yield were estimated using the following formula [75]:

(6) $$\mathrm{Essential}\;\mathrm{oil}\;\mathrm{content}\;\left(\%\right)=\frac{\mathrm{Extracted}\;\mathrm{EO}\;\left(g\right)}{50\;g\;\mathrm{of}\;\mathrm{ajwain}\;\mathrm{seed}}\times 100$$

(7) $$\mathrm{Essential}\;\mathrm{oil}\;\mathrm{yield}\;({\mathrm{kg}\;\mathrm{ha}}^{-1})=\mathrm{seed}\;\mathrm{yield}\;({\mathrm{ka}\;\mathrm{ha}}^{-1})\times \mathrm{Essential}\;\mathrm{content}\;\left(\%\right)$$

### 4.4. Leaf Traits and Biochemical Analyses

#### 4.4.1. Determination of Nitrogen, Phosphorus, and Potassium Concentration

Ajwain leaves were ground, digested, and combusted for 4 h at 500 °C to determine nutrient uptake. The ash particles (5 mg) were digested in 1 mL of 2 N HCl and then separated using Whatman filter paper (grade 42). The phosphorus uptake was determined calorimetrically using the vanadomolybdate method, which involved the production of the yellow-colored complex of the unreduced vanadomolybdophosphoric heteropoly acid, stabilized in an HNO_3_ medium. The color intensity was measured at 470 nm using a Spectronic 20 colorimeter [76,77]. Potassium uptake was determined using a flame photometer [76,77]. Nitrogen (N) was analyzed using the Kjeldahl technique [78].

#### 4.4.2. Relative Water Content (RWC)

The RWC was calculated according to Equation (5) [79]:

(8) $$\mathrm{RWC}\;\left(\%\right)=\frac{\left(\mathrm{FW}-\mathrm{DW}\right)}{\left(\mathrm{TW}\;-\mathrm{DW}\right)}\times 100$$

Following the measurement of fresh weight (FW), the leaves were immersed in distilled water for 16 to 18 h. After immediately blotting the saturated samples to remove any water residues, the saturated weight (TW) was measured. The samples were oven-dried for 24 h at 70 °C to determine their dry weight (DW).

#### 4.4.3. Chlorophyll a and b Content

Fresh leaf tissue (0.5 g) collected at the full flowering stage was pulverized in a nitrogen solution, mixed with 10 mL of 80% acetone, and homogenized by centrifugation at 4000 rpm for 15 min. The procedure was repeated until a white pellet was obtained. Subsequently, the extracted pigments were quantified using a spectrophotometer, allowing for the accurate measurement of chlorophyll a and b contents [80].

#### 4.4.4. Total Soluble Sugars

The total soluble sugar content in leaves was evaluated through the phenol–sulfuric acid method. Leaf tissue (0.5 g) was powdered in a mortar using a nitro-gen solution, mixed with ethanol, and combined with 5% phenol. Next, 5 mL of 98% sulfuric acid was added to the mixture and incubated for 1 h before measuring the absorption of the solution at 625 nm using a spectrophotometer [81]. A standard curve (R^2^ = 0.99, ranging from 0 to 80 mg L^−1^) was generated by plotting solutions with varying D-glucose concentrations. The results were established via the standard curve and expressed as mg g^−1^ of FW [82].

#### 4.4.5. Proline Content

Leaf proline content was determined using the ninhydrin colorimetric method. Leaf tissue (0.5 g) was finely powdered in a mortar using liquid nitrogen, homogenized in 10 mL of 3% sulfosalicylic acid solution, and centrifuged at 4000 rpm for 15 min to obtain a clear supernatant. Then, a glacial acetic acid solution of pro-line ninhydrin acid was prepared (1:1:1, *v*:*v*:*v*) and equilibrated at 100 °C for 1 h to facilitate the reaction between proline and ninhydrin, forming a chromophore. The reaction was stopped by rapidly cooling the solution in an ice bath. To develop the chromophore, 4 mL of toluene was added to the reaction mixture, enabling the extraction of the chromophore into the organic phase. Finally, the absorbance of the samples was measured at 515 nm using a spectrophotometer [82]. L-proline was used as standard to make a reference curve (R^2^ = 0.99, ranging from 0 to 150 mg L^−1^). Proline content was established from the reference curve and expressed as mg g^−1^ of FW [82,83].

#### 4.4.6. Enzyme Extractions and Assays

To measure antioxidant enzyme activity, 100 mg of fresh material was finely powdered and treated with 2 mL of a 0.1 M KH_2_PO_4_ buffer containing 5% polyvinylpyrrolidone (PVP) at pH 6. The extracts were centrifuged at 15,000 rpm for 30 min at 3 °C, and the clear supernatant was used to determine before the protein content by Bradford method [84] and, then, the specific activity of the enzyme as reported below.

Catalase (CAT) activity was measured at 240 nm through the variation in hydrogen peroxide (H_2_O_2_) concentration. The reaction mixture contained 1.9 mL of 50 mM K_3_PO_4_, which was buffered at a pH of 7, 10 mM of H_2_O_2_, and 0.2 mL of enzyme extract. Enzymatic activity was then measured in 60 s per mg of protein based on absorption variations [84].

Superoxide dismutase (SOD) activity was measured at 560 nm to minimize the photochemical loss of nitroblue tetrazolium (NBT), as described by Beyer and Fridovich [85]. One unit of SOD was defined as the enzyme amount required to inhibit a 50% decrease in NBT.

The ascorbate peroxidase (APX) activity was measured using a reaction mixture containing 1 mL of 0.5 mM ascorbic acid and 1 mL 100 mM K_3_PO_4_—buffered at a pH of 7, 100 μL of enzyme extract and 0.1 mL of H_2_O_2_ 0.1 mM. The absorption was then read at 290 nm.

#### 4.4.7. Total Polyphenol and Flavonoid Leaf Content

The total polyphenol content was assessed using the Folin–Ciocalteau method [86,87]. A total of 1600 μL of sterilized water and 10 μL of methanolic extracts were mixed and then subjected to treatment with 200 μL of Folin–Ciocalteau reagent (10% *v*:*v*) for 5 min at 25 °C. After adding 200 μL of 7.5% NaCO_3_, the reaction mix was preserved at 25 °C in darkness for 30 min. To estimate the polyphenol concentration, a UV/Visible spectrophotometer (DB-20/DB-20S) was utilized to evaluate the sample’s absorbance at 760 nm. A calibration curve (R^2^ = 0.9556) built with gallic acid (3,4,5 trihydroxybenzoic acid), ranging from 0 to 500 mg L^−1^, was used to determine the TPC. The results are expressed as milligrams of gallic acid equivalent per gram of the dry weight sample (mg GAE g^−1^ of DW).

The total flavonoid content of flower extracts was determined through the aluminum chloride-based colorimetric method. In summary, 30 μL of the extract was combined with 150 μL of sodium nitrate (5% *w*/*v*) and 3 mL of 10% *w*/*v* aluminum chloride hexahydrate. The reaction mix was incubated for 5 min. After that, 1 mL of NaOH (1.0 M) was incorporated, and then, the purified water was properly diluted. The solution’s absorbance was read at 510 nm in a UV spectrophotometer implementing a 30 min darkness period of incubation at 25 °C [86,87]. The TFC was determined using a quercetin standard curve (R^2^ = 0.9922) ranging from 0 to 10 mg L^−1^. The results are expressed as milligrams of equivalents for each gram of sample of dry weight (mg QE g^−1^ of DW).

### 4.5. Data Analysis

A two-year analysis of variance (ANOVA) was conducted applying the general linear model (GLM) (SAS 9.1.3), combining the findings of the years 2022–2023. The impacts of the irrigation regime, the stress modifier nanoparticles, and their interactions were assessed by two-way ANOVA. The LSD test was used for comparing means at *p* < 0.05.

## 5. Conclusions

The present study may be considered pivotal in testing the effect of foliar spraying of stress modifiers, specifically selenium, chitosan, melatonin, and humic acid on the ajwain plant subjected to water scarcity.

Melatonin and chitosan were more effective than humic acid and selenium in sustaining yields of seed, fixed oil, and essential oil. Melatonin alleviated the adverse impacts of water deficit by improving osmotic adjustments. This allows us to maintain a higher leaf relative water content, as well as chlorophyll and nutrient concentration, antioxidant activity, and essential oil production.

Encouraging results were also obtained with chitosan, suggesting that both melatonin and chitosan considerably help plant cope with water stress through increasing proline concentration and osmotic regulation.

Melatonin and chitosan have shown great potential in enhancing the growth, physiological characteristics, and essential and fixed oil production in ajwain plants. Their use has been proven to effectively mitigate the impact of water stress, thus supporting sustainable ajwain cultivation in semi-arid and arid regions of Iran. While there are costs associated with purchasing and applying these biostimulants, our research has demonstrated that the benefits, such as increased crop yields, the promotion of organic farming, and the reduction in negative environmental impacts from chemical fertilizers, far outweigh the initial investment. This study marks an important initial exploration of the application of these modifiers as an effective nature-based solution. However, further research is needed to fully understand the mechanisms underlying these positive effects.

## Figures and Tables

**Table 1:** Analysis of variance and mean comparison of nutritional traits of ajwain plants in response to different irrigation regimes (Irs), stress modifier application (M), and their interaction (Ir × M) conducted during two consecutive years, 2022–2023 (Y). The asterisks ** indicate the significance at 1% probability levels according to LSD test (*p* < 0.05), NS: not significant differences. Different letters indicate significant differences among diverse treatments.

Source of Variation	df	BY	SY	FO	FOY	EO	EOY	N	P	K
Year (Y)	1	632,488 **	18,208 **	0.01 ^NS^	370.43 **	0.0008 ^NS^	14.99 **	90.70 **	0.42 **	26.35 **
Rep (Y)	4	14,060	69.35	0.03	6.99 ^NS^	0.001 ^NS^	0.17	0.05	0.0003	0.02
(Ir)	2	13,509,080 **	194,134 **	78.87 **	14,290 **	0.76 **	193.22 **	137.04 **	0.88 **	72.13 **
Y × Ir	2	8122 ^NS^	41.81 ^NS^	0.02 ^NS^	6.68 ^NS^	0.001 ^NS^	0.03 ^NS^	0.04 ^NS^	0.0003 ^NS^	0.04 ^NS^
(M)	4	1,618,004 **	6941 **	5.43 **	722.3 **	0.22 **	48.95 **	18.92 **	0.06 **	8.14 **
Y × M	4	454 ^NS^	51.33 ^NS^	0.02 ^NS^	2.97 ^NS^	0.0002 ^NS^	0.09 ^NS^	0.12 ^NS^	0.0002 ^NS^	0.06 ^NS^
Ir × M	8	1,280,608 **	8599 **	1.42 **	386.3 **	0.003 **	0.45 **	3.84 **	0.01 **	1.83 **
Y × Ir × M	8	718 ^NS^	77.59 ^NS^	0.03 ^NS^	6.14 ^NS^	0.0002 ^NS^	0.05 ^NS^	0.04 ^NS^	0.0002 ^NS^	0.02 ^NS^
Error	56	27,838	79.77	0.04	7.00	0.0006	0.12	0.10	0.0004	0.06
**Year**										
2022		2758 ± 52.92 a	601.6 ± 10.23 b	14.9 ± 1.49 a	90.8 ± 4.80 b	3.00 ± 0.17 a	18.1 ± 2.58 b	22.0 ± 2.12 b	2.45 ± 0.18 b	15.2 ± 1.51 b
2023		2690 ± 72.03 a	630.0 ± 9.300 a	14.9 ± 1.47 b	94.9 ± 4.21 a	2.98 ± 0.16 a	18.9 ± 2.62 a	24.1 ± 2.07 a	2.59 ± 0.15 a	16.2 ± 1.47 a
**Irrigation Regime**										
FC100%		3405 ± 93.51 a	693.5 ± 12.55 a	16.5 ± 0.51 a	114 ± 4.50 a	2.87 ± 0.11 c	19.8 ± 1.73 b	25.3 ± 1.51 a	2.68 ± 0.09 a	17.3 ± 0.87 a
FC75%		2979 ± 39.26 b	620.9 ± 10.10 b	15.0 ± 0.67 b	93.5 ± 5.74 b	3.18 ± 0.10 a	20.1 ± 1.61 a	22.8 ± 1.64 b	2.54 ± 0.10 b	15.6 ± 1.03 b
FC50%		1790 ± 19.53 c	532.9 ± 8.540 c	13.2 ± 0.73 c	70.7 ± 4.30 c	2.95 ± 0.11 b	15.6 ± 1.44 c	21.0 ± 1.44 c	2.34 ± 0.11 c	14.2 ± 0.93 c
**Modifiers**										
MEN		3056 ± 16.05 a	642 ± 5.68 a	15.7 ± 1.08 a	101 ± 6.68 a	3.13 ± 0.13 a	20.4 ± 2.37 a	24.2 ± 1.94 a	2.60 ± 0.14 a	16.5 ± 0.18 a
CHN		2960 ± 58.24 a	630 ± 4.59 b	15.2 ± 1.03 b	96.8 ± 4.59 b	3.09 ± 0.12 b	19.7 ± 2.07 b	23.9 ± 1.92 b	2.56 ± 0.12 b	16.3 ± 0.32 b
HUC		2756 ± 44.84 b	610 ± 9.30 c	14.8 ± 1.59 c	91.2 ± 7.50 c	2.99 ± 0.16 c	18.4 ± 2.41 c	23.1 ± 2.32 c	2.52 ± 0.18 c	15.7 ± 1.56 c
SEM		2510 ± 32.10 c	602 ± 7.33 d	14.5 ± 1.53 d	88.8 ± 6.45 d	2.92 ± 0.14 d	17.8 ± 2.20 d	22.2 ± 2.30 d	2.47 ± 0.17 d	15.2 ± 1.70 d
CON		2341 ± 58.99 d	595 ± 9.24 e	14.3 ± 1.74 e	85.8 ± 7.39 e	2.86 ± 0.13 e	16.2 ± 1.93 e	21.9 ± 2.33 e	2.46 ± 0.21 e	14.9 ± 1.64 e
CV (%)		5.77	1.45	1.37	2.85	0.87	1.88	1.41	0.82	1.58

BY (biological yield) (kg ha^−1^); SY (seed yield) (kg ha^−1^); FO (fixed oil) (%); FOY (fixed oil yield) (kg ha^−1^); EO (essential oil) (%); EOY (essential oil yield) (kg ha^−1^); N, nitrogen (mg g^−1^); P, phosphorus (mg g^−1^); K, potassium (mg g^−1^); MEN, melatonin; HUC, humic acid; CHN, chitosan; SEM, selenium; and CON, control.

**Table 2:** Analysis of variance and mean comparison of eco-physiological traits of ajwain plants in response to different irrigation regimes (Irs), stress modifier application (M), and their interaction (Ir × M) conducted during two consecutive years, 2022–2023 (Y). The asterisks ** indicate the significance at 1% probability levels according to LSD test (*p* < 0.05), NS: not significant differences. Different letters indicate statistically significant differences among treatments.

Source of Variation	df	RWC	Chl a	Chl b	TSS	Pro	APX	SOD	CAT	TFC	TPC
Year (Y)	1	248.93 **	0.23 **	0.09 **	46.32 **	0.09 ^NS^	0.02 **	4.34 ^NS^	0.05 ^NS^	0.29 **	59.17 **
Rep (Y)	4	0.32	0.009	0.004	0.59	0.01	0.001	0.32 ^NS^	0.003	0.004	0.76
(Ir)	2	1946.39 **	7.06 **	2.32 **	260.05 **	24.53 **	0.27 **	2941.11 **	27.87 **	2.97 **	315.44 **
Y × Ir	2	0.23 ^NS^	0.0004 ^NS^	0.004 ^NS^	0.08 ^NS^	0.007 ^NS^	0.0004 ^NS^	7.75 ^NS^	0.09 ^NS^	0.001 ^NS^	0.10 ^NS^
(M)	4	387.06 **	0.93 **	0.23 **	28.05 **	0.2.80 **	0.05 **	612.36 **	6.16 **	0.42 **	37.85 **
Y × M	4	0.20 ^NS^	0.003 ^NS^	0.001 ^NS^	0.007 ^NS^	0.0004 ^NS^	0.0001 ^NS^	2.48 ^NS^	0.02 ^NS^	0.0009 ^NS^	0.01 ^NS^
Ir × M	8	244.13 **	0.32 **	0.18 **	10.01 **	0.31 **	0.03 **	382.36 **	3.29 **	0.13 **	11.24 **
Y × Ir × M	8	0.51 ^NS^	0.0002 ^NS^	0.001 ^NS^	0.004 ^NS^	0.0006 ^NS^	0.0001 ^NS^	3.37 ^NS^	0.03 ^NS^	0.0003 ^NS^	0.005 ^NS^
Error	56	6.91	0.008	0.004	0.25	0.02	0.0008	6.91	0.06	0.002	0.32
**Year**											
2022		64.3 ± 9.50 b	2.46 ± 0.50 b	1.59 ± 0.28 b	15.6 ± 2.92 a	3.56 ± 0.85 a	1.12 ± 0.12 a	48.7 ± 12.2 a	3.26 ± 1.19 a	1.90 ± 0.32 a	20.2 ± 3.23 a
2023		67.6 ± 9.29 a	2.57 ± 0.47 a	1.66 ± 0.30 a	14.2 ± 2.84 b	3.50 ± 0.86 a	1.08 ± 0.10 a	48.3 ± 10.9 a	3.21 ± 1.06 a	1.79 ± 0.29 b	18.6 ± 3.14 b
**Irrigation Regime**											
FC100%		74.2 ± 6.15 a	2.99 ± 0.31 a	1.90 ± 0.15 a	11.8 ± 1.95 c	2.55 ± 0.55 c	1.02 ± 0.09 c	38.9 ± 7.41 c	2.28 ± 0.67 c	1.52 ± 0.21 c	16.0 ± 2.13 c
FC75%		65.5 ± 8.49 b	2.54 ± 0.35 b	1.63 ± 0.23 b	15.2 ± 1.89 b	3.69 ± 0.43 b	1.07 ± 0.09 b	47.9 ± 10.2 b	3.21 ± 1.00 b	1.85 ± 0.22 b	19.8 ± 2.14 b
FC50%		58.1 ± 5.71 c	2.02 ± 0.17 c	1.35 ± 0.14 c	17.7 ± 1.20 a	4.34 ± 0.21 a	1.20 ± 0.07 a	58.7 ± 6.74 a	4.21 ± 0.68 a	2.15 ± 0.14 a	22.5 ± 1.40 a
**Stress Modifiers**											
MEN		71.2 ± 6.99 a	2.80 ± 0.59 a	1.75 ± 0.35 a	16.7 ± 2.88 a	3.98 ± 0.80 a	1.16 ± 0.11 a	55.2 ± 7.05 a	3.92 ± 0.72 a	2.08 ± 0.30 a	21.7 ± 3.15 a
CHN		68.3 ± 11.06 b	2.73 ± 0.50 b	1.72 ± 0.30 a	15.6 ± 1.78 b	3.80 ± 0.86 b	1.13 ± 0.16 b	51.2 ± 15.1 b	3.55 ± 1.39 b	1.91 ± 0.19 b	19.9 ± 2.01 b
HUC		66.5 ± 6.02 b	2.41 ± 0.39 c	1.59 ± 0.22 b	14.4 ± 2.76 c	3.59 ± 0.99 c	1.11 ± 0.07 bc	49.3 ± 6.68 c	3.32 ± 0.67 c	1.78 ± 0.70 c	17.7 ± 4.08 c
SEM		64.7 ± 7.67 c	2.37 ± 0.35 c	1.55 ± 0.24 b	14.2 ± 2.52 c	3.26 ± 0.62 d	1.09 ± 0.05 c	47.5 ± 10.3 c	3.00 ± 0.98 d	1.75 ± 0.24 d	18.6 ± 2.87 c
CON		58.9 ± 9.57 d	2.29 ± 0.38 d	1.50 ± 0.25 c	13.6 ± 2.67 d	3.01 ± 0.62 e	1.01 ± 0.10 d	39.4 ± 11.1 d	2.37 ± 1.11 e	1.70 ± 0.33 e	18.2 ± 2.88 d
CV (%)		3.98	3.68	3.92	3.36	4.56	2.57	5.41	8.13	2.63	2.92

RWC, relative water content (%); Chl a and b, chlorophyll a and b content (mg g^−1^ FW); TSS, total soluble sugars (mg g^−1^ FW); Pro, proline (mg g^−1^ FW); APX, ascorbate peroxidase activity (µmol min^−1^ g^−1^ FW); SOD, superoxide dismutase activity (µmol min^−1^ g^−1^ FW); CAT, catalase activity (µmol min^−1^ g^−1^ FW); TFC, total flavonoid content (mg g^−1^ DW); TPC, total phenol content (mg g^−1^ DW); MEN, melatonin; HUC, humic acid; CHN, chitosan; SEM, selenium; and CON, control.

## Data Availability

Data are available from the corresponding author upon reasonable request.
